# Best Practices for the Use of High-Frequency Ultrasound to Guide Esthetic Filler Injections—Part 3: Lower Third of the Face

**DOI:** 10.3390/diagnostics15070921

**Published:** 2025-04-02

**Authors:** Roberta Vasconcelos-Berg, Stella Desyatnikova, Paula Bonavia, Alexander Navarini, Maria Cristina Chammas, Rosa Sigrist

**Affiliations:** 1 Margarethenklinik—University Hospital of Basel, 4051 Basel, Switzerland; paulavalentina.bonavia@usb.ch (P.B.); alexander.navarini@usb.ch (A.N.); 2 The Stella Center for Facial Plastic Surgery, Seattle, WA 98101, USA; stella@doctorstella.com; 3 Department of Radiology, School of Medicine, University of São Paulo, São Paulo 05403-010, Brazil; mcchammas@hotmail.com (M.C.C.); rm@sigrist.com.br (R.S.)

**Keywords:** filler, hyaluronic acid, guided injections, lips, jawline, pre-jowl, marionette line, perioral, chin

## Abstract

**Background:** The lower third of the face plays a crucial role in overall facial harmony, and age-related volume loss in areas such as the pre-jowl region, labiomental folds, and lips can significantly impact esthetic appearance. High-resolution ultrasound is helpful for identifying key structures, such as the facial artery, parotid gland, and masseter muscle, which are critical to avoid during filler injections. **Objectives:** This article, the final installment in a three-part series on ultrasound-guided facial injections, provides an in-depth analysis of the sonographic anatomy of the lower face, including the mandibular, marionette, and chin regions. **Methods**: This article outlines step-by-step techniques for ultrasound-guided filler procedures, with a focus on the importance of pre- and intra-procedural scanning to ensure safe and accurate filler placement. **Results:** By employing techniques like “scan before injecting” and “scan while injecting”, injectors aim to reduce risks such as vascular occlusion, muscle injection, and skin necrosis. **Discussion:** The use of ultrasound guidance in these regions enhances both esthetic outcomes and patient safety, providing optimal results while minimizing complications. With continued advancements, ultrasound-guided injections will become increasingly precise, enabling more targeted and safer treatments in the lower face.

## 1. Introduction

The lower face holds significant importance for facial esthetics. When balanced with the other facial thirds, it enhances overall harmony, making the face more attractive and visually appealing. Additionally, the lower face plays a critical role in defining sexual dimorphism. A squared jawline, with a larger bigonial width [1], and a broader chin [2] are features typically associated with male individuals [3] and are often perceived as more attractive when accentuated [4].

As individuals age, the lower face undergoes distinct anatomical changes that are part of the broader multifactorial process of facial aging [5]. This process affects all layers of the face, including the skin, soft tissue, and underlying skeletal structure, progressing at varying rates among individuals. The skin becomes thinner, subcutaneous fat redistributes, and the bones of the face experience remodeling and resorption. Specifically, in the lower face, the mandible undergoes anterior and inferior resorption, a process exacerbated by tooth loss. The mandibular angle increases over time due to resorption along its lower border, while the chin shifts anteriorly and becomes shorter. Fat compartments in the superior and inferior mandibular regions undergo atrophy, and the weakening of the mandibular septum leads to fat pad displacement towards the neck.

These anatomical changes, combined with skin thinning, contribute to the loss of jawline definition and the development of jowls. The situation is further aggravated by the downward pull of soft tissues by muscles such as the depressor labii inferioris, depressor anguli oris, and platysma. Furthermore, individuals with a congenitally small or recessed chin are predisposed to an earlier loss of jawline definition, emphasizing the importance of addressing these structural features in esthetic treatments [5].

The lower third of the face also includes the lips and the perioral region, which play a key role in facial esthetics and attractiveness. These areas also undergo significant changes with aging, including loss of volume and elasticity.

Hyaluronic acid fillers and biostimulators are essential tools for minimally invasive rejuvenation of the lower third of the face. To optimize treatment outcomes, it is crucial to apply each type of product to the correct anatomical layer. High G prime fillers are typically placed in deeper planes, such as the supraperiosteal layer, while lower G prime fillers are more suited for superficial planes. Additionally, injecting the product above or below specific muscles may achieve a myomodulatory effect [6,7].

Care must also be taken to avoid injecting into critical anatomical structures, such as blood vessels, muscles, or glands, to prevent complications. In this context, ultrasound imaging is increasingly recognized as a valuable tool for guiding injectable esthetic procedures. The growing need for protocols to assist injectors in effectively utilizing this technology highlights its importance in advancing patient safety and outcomes.

In recent years, high-frequency ultrasound has proven to be a valuable tool in esthetic dermatology. One of its key applications is assisting with filler and biostimulator injections, allowing for greater precision. When used during the procedure, it enables the injector to visualize anatomical structures in real time, determine the optimal injection plane, and avoid blood vessels or other high-risk areas.

This article is the third in a series [8,9] dedicated to exploring ultrasound-guided injection techniques for the three facial thirds, providing an in-depth analysis of methods and anatomical considerations to enhance precision, safety, and esthetic outcomes.

## 2. Materials and Methods

This narrative review is based on the authors’ extensive experience with high-frequency DUS-guided injections in the lower face. The techniques and recommendations outlined in this paper represent the authors’ personal approach and should be interpreted accordingly.

The ultrasound devices used in this study included the LOGIQ E10 and LOGIQ e (GE Healthcare, Waukesha, WI, USA), equipped with linear probes operating at 6–24 MHz and 8–18 MHz, the Venue Go (GE Healthcare, Waukesha, WI, USA) with a linear probe range of 4–20 MHz, and the ACUSON Sequoia (Siemens Medical Solutions, Mountain View, CA, USA) featuring a linear probe range of 6–18 MHz.

Before performing the procedures, the treatment area was thoroughly disinfected, and the linear probe was covered with a sterile transparent dressing (Opsite^®^, Smith & Nephew Medical, Suzhou, China) following a previously described protocol by the authors’ team [10].

Injections were administered using either a blunt-tipped cannula (22 G or 25 G) or a sharp needle (27 G or 30 G), depending on the specific requirements of the treatment.

As previously described by the authors [10], ultrasound-guided injection techniques were categorized into two primary approaches:

- **Scan before injecting:** The treatment area is scanned immediately prior to the injection to identify the location of vascular structures. The paths of the main arteries can be marked on the patient’s skin to guide the procedure.
- **Scan while injecting:** In this method, the cannula is tracked in real time using ultrasound as it is guided to the target anatomical layer, ensuring that vascular structures are avoided.

Regarding the positioning of the ultrasound probe relative to the cannula, both in-plane and out-of-plane techniques were utilized. The in-plane technique is generally favored because it provides a clear, real-time view of the cannula’s full length. However, if the cannula is positioned near a blood vessel, the out-of-plane technique can be used to verify that the cannula tip is not within the vessel.

## 3. Results

### 3.1. Mandibular Region (Posterior and Anterior)

#### 3.1.1. Sonographic Anatomy

**Figure 1:**
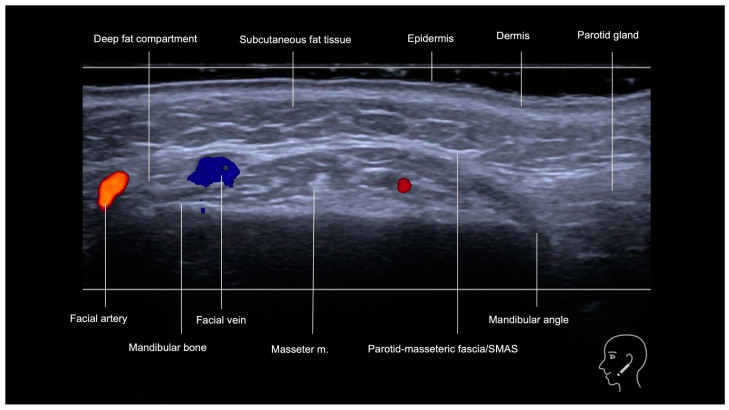
A gray-scale ultrasound of the mandibular region (jawline and angle of the jaw) in a transverse view, using an 18 MHz probe. Anterior to the masseter muscle, the deep fat compartment is visualized alongside two key vascular structures. The facial vein, positioned more posteriorly, is easily compressible under the ultrasound transducer, while the facial artery, located anteriorly, is pulsatile and typically tortuous as it courses toward the oral angle.

The mandibular region is divided didactically into posterior and anterior. Ultrasound imaging of the posterior mandibular region, near the angle of the jaw, which corresponds to the jawline, reveals that the layers in this area consist of the skin, subcutaneous tissue, parotid gland—which may contain lymph nodes—the masseter muscle, and the mandibular bone, as shown in Figure 1.

The subcutaneous tissue, appearing as a hypoechoic layer with tortuous hyperechoic septa, can vary in thickness in this region. An anterior extension of the parotid gland or the presence of an accessory parotid gland (observed in approximately 20% of the population) [11] can further reduce the thickness of this subcutaneous layer [12]. The tail of the parotid gland extends inferiorly toward the sternocleidomastoid muscle [11] and should be considered when performing injections at the angle of the jaw. The masseter muscle, a hypoechoic rectangular muscle, is characterized by hyperechoic tendinous aponeuroses and tendons.

The vascularization of the posterior mandibular region typically lacks large vessels; however, care should be taken with small subcutaneous vessels, which can be visualized with Doppler ultrasound.

**Figure 2:**
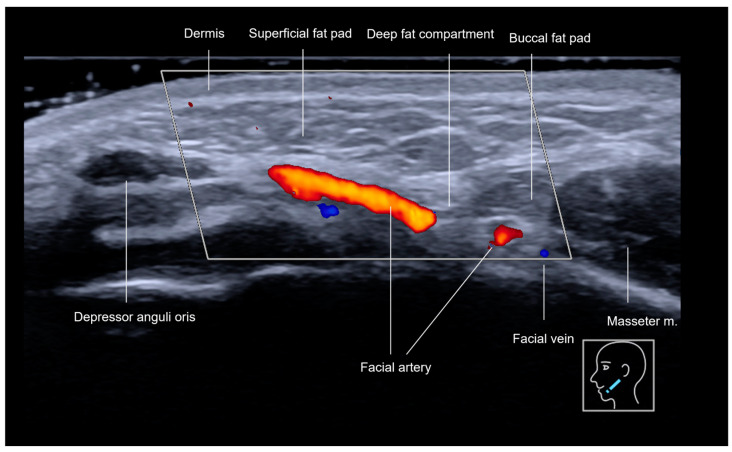
A Doppler ultrasound of the anterior mandibular region in a transverse view, using an 18 MHz probe.

**Figure 3:**
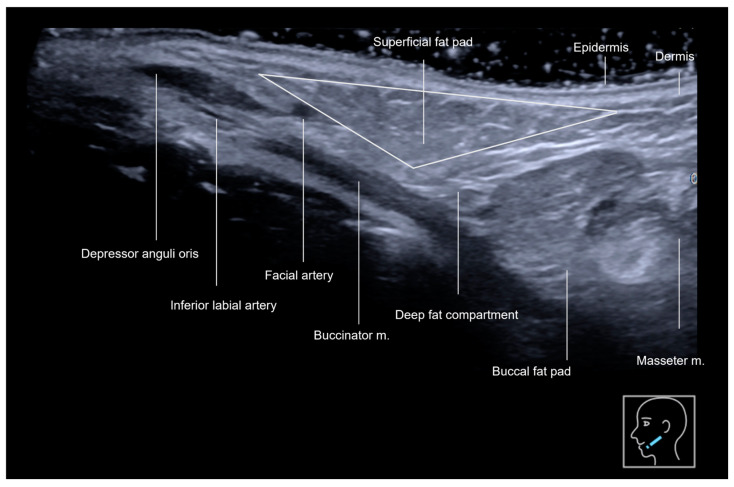
A gray-scale ultrasound of the anterior mandibular region layers in a transverse view, obtained with an 18 MHz probe. The triangular superficial fat compartment will, over time, contribute to the formation of the jowl.

The anterior mandibular region (Figure 1 and Figure 2), located anteriorly to the masseter muscle, encompasses the deep fat pad which contains two key vascular structures. The facial vein, positioned more posteriorly, is easily compressible under the ultrasound transducer, while the facial artery, located anteriorly, is pulsatile and it courses toward the oral angle (Figure 2). The facial artery gives rise to the inferior labial artery, which runs beneath the depressor anguli oris muscle (Figure 2). In this region, the superficial fat pad forms an inverted triangular configuration, corresponding to the thickest part of the superficial fat pad (Figure 3). Over time, this area contributes to the formation of the jowl as it loses volume and elasticity. The pre-jowl is the area located anteriorly to the jowl. These anatomical landmarks are essential in ultrasound assessments, facilitating safe procedural guidance and helping to prevent vascular complications.

#### 3.1.2. Ultrasound-Guided Filling Techniques for the Posterior Mandibular Region (Jawline)

**Technique 1:** Deep injections of the mandibular angle using a needle

To enhance the mandibular angle and increase the bigonial width—often desired in facial masculinization procedures, changes in facial shape, or for better definition of the jawline area—hyaluronic acid can be injected in bolus form into the mandibular angle.

**Figure 4:**
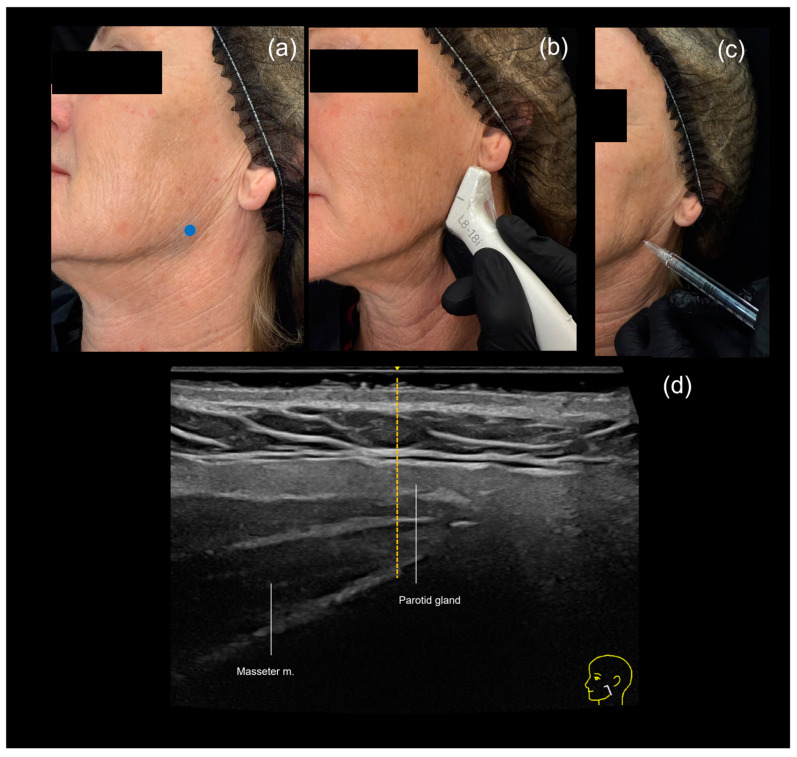
An ultrasound-guided filler injection of the mandibular angle using the “scan before injecting” technique. (**a**) The treatment area is marked (blue dot). (**b**) The marked area is scanned, and the depth of the masseter muscle is measured. (**c**) The site is carefully injected with a 27 G needle following aspiration. (**d**) Ultrasound measurement of the masseter muscle (orange dotted line) performed with a linear transducer L4-20.

The technique consists of palpating the natural curve of the mandible where the ramus meets the body, then injecting a high G prime filler using a needle, typically 27 G. When performing this procedure with ultrasound guidance, we recommend using the “scan before injecting” technique. The injection point is marked (Figure 4a) and then scanned using Doppler ultrasound (Figure 4b).

While this region does not typically contain large vessels, the masseter muscle is often present, and intramuscular injections can cause post-procedure pain and restricted chewing. To avoid injecting into the masseter, the depth of the muscle can be measured (Figure 4d) at the injection point, and the needle length can be adjusted accordingly, avoiding the use of needles that are shorter than necessary. Then, the filler is injected and the needle is advanced until resistance is felt upon contact with the bone, and then a bolus of approximately 0.5 mL is injected after aspiration (Figure 4c).

**Technique 2:** Injection of the jawline with a blunt cannula

To contour and define the jawline area, our group prefers using cannulas, following a linear path along the region to be treated. The jowl, due to its excess volume, should not be filled. The entry point for the cannula can be either at the mandibular angle or near the jowl. In this region, the anatomical structures to avoid include the facial artery, which is generally located anterior to the anterior masseter border and is deep. It coincides with the jowl. Other relevant structures along the jawline include the masseter muscle and the parotid gland. Therefore, it is recommended to make the entry point and keep the cannula in the subcutaneous plane.

**Figure 5:**
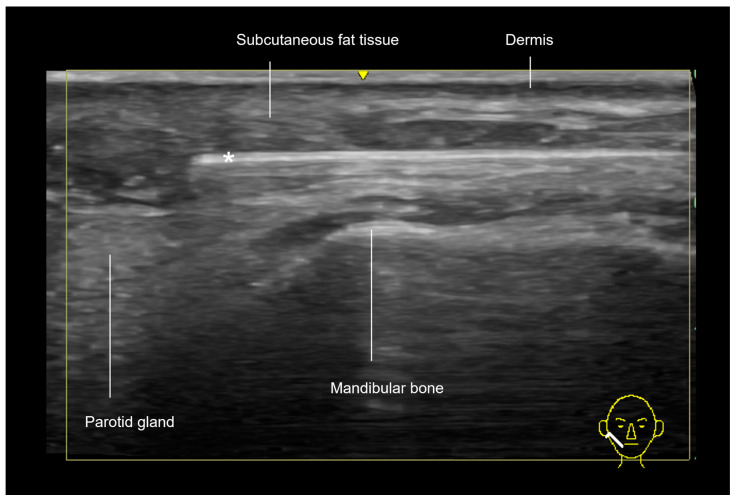
An ultrasound-guided filler injection along the jawline using the “scan while injecting” technique, demonstrating a 22 G cannula (*) positioned within the subcutaneous fat compartment in an in-plane approach. Doppler ultrasound, transverse view with an 18 MHz probe, shows no visible arteries.

Ultrasound can assist in ensuring that the cannula is not in contact with the facial artery and is in the correct plane, avoiding the aforementioned structures. For this, we use the “scan while injecting” technique, where after inserting the cannula, we check its position with our in-plane probe to confirm correct placement (Figure 5).

#### 3.1.3. Ultrasound-Guided Filling Techniques of the Anterior Mandibular Region (Pre-Jowl)

The pre-jowl region is often filled to create continuity along the jawline, resulting in a lengthening effect on the face. Additionally, filling this area can help to camouflage the excess volume of the jowl, which naturally occurs with aging. Treating the pre-jowl area can also be desirable in the context of chin augmentation, as it helps to increase the width of the chin. Figure 2 and Figure 3 illustrate the ultrasound anatomy of this area. In this region, the highest-risk structure is the facial artery, which emerges from beneath the mandible anterior to the masseter muscle and continues its course beneath the depressor anguli oris muscle in the same area.

**Technique 1:** Deep injections with a needle

**Figure 6:**
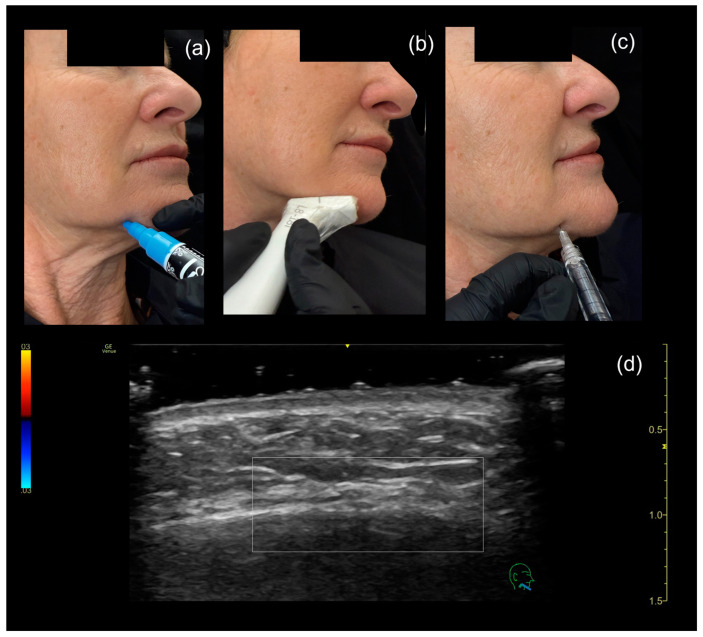
An ultrasound-guided filler injection of the pre-jowl area with a needle using the “scan before injecting” technique. (**a**) The treatment area is marked. (**b**) The marked area is scanned. (**c**) The point is carefully injected with a 27 G needle. (**d**) An US-Doppler image obtained with a linear L4-20 transducer showing no detectable blood flow at the supraperiosteal level in the pre-jowl area (white rectangle).

In some cases, needle-based filler injections are preferred in this region due to their greater precision. In such instances, the supraperiosteal plane is chosen, using a high G prime filler with a strong lifting capacity. Ultrasound guidance is primarily used to avoid the facial artery. The injection point(s) should be marked on the skin in advance and scanned using Doppler ultrasound (“scan before injecting”). After confirming the absence of supraperiosteal vessels in the area, the filler is injected as planned, using a 27 G needle, supraperiosteally, and perpendicular to the skin (Figure 6).

**Technique 2:** Injection with a blunt cannula

**Figure 7:**
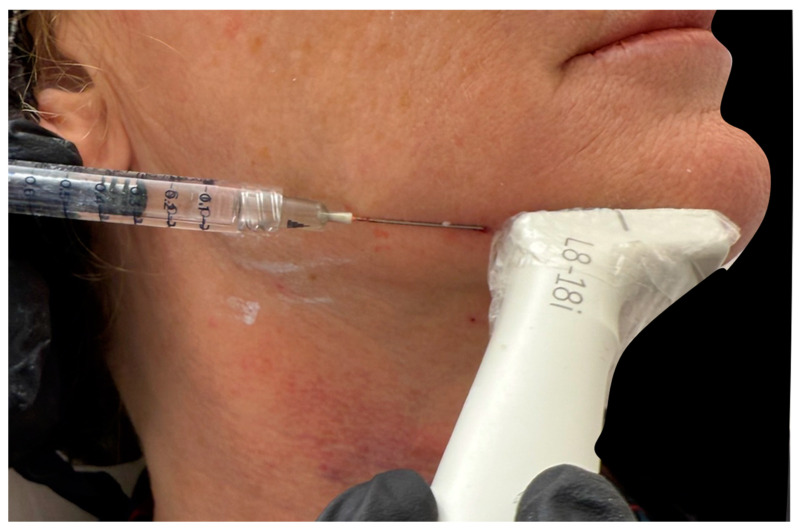
An ultrasound-guided filler injection of the pre-jowl using the “scan while injecting” technique. Treatment showing the cannula and ultrasound probe in an in-plane alignment.

Similarly to the jawline region, the pre-jowl can also be filled using a cannula, with the subcutaneous plane being the preferred approach. An entry point is made either in the jowl or the lateral chin area, and the cannula is inserted in the desired direction, aligning with the pre-jowl line. The preferred technique for guided filler injection is “scan while injecting,” allowing for verification of the cannula’s position and ensuring that the cannula tip does not come into contact with any vascular structures (Figure 7).

### 3.2. Labiomandibular Folds or Marionette Lines

Labiomandibular or melomental folds, commonly referred to as marionette lines, become more pronounced with aging due to a combination of bone resorption in the maxilla and mandible, gravitational descent, and volume loss in the deep fat layer beneath the depressor anguli oris muscle. Additional factors include the tethering effect of the mandibular ligament, sagging of excess skin and connective tissue, and the drooping of jowl and buccal fat. Together, these changes deepen the wrinkle and extend it toward the jawline [13].

**Figure 8:**
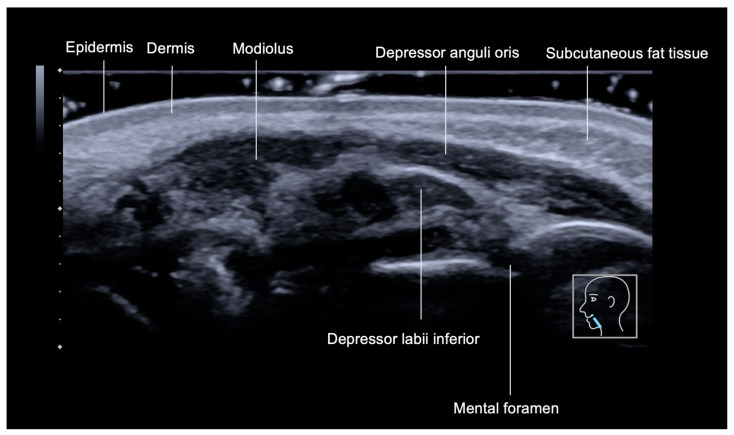
An ultrasound of the layers of the labiomental fold (marionette line) in an oblique view, obtained with an 18 MHz probe.

In this area, ultrasound reveals several distinct layers (Figure 8): the skin (epidermis and dermis), subcutaneous fat tissue, the depressor anguli oris muscle, and the depressor labii inferioris muscle. The mental foramen, where the mental nerve and vessels emerge, appears on ultrasound as a gap within the mandibular bone. These structures serve as important landmarks for evaluating the anatomy of this region, particularly during esthetic procedures or treatments involving this area.

#### Ultrasound-Guided Filling Techniques of the Marionette Line

In this area, it is crucial to avoid the inferior labial artery, which runs beneath the *depressor anguli oris* (DAO) muscle, as well as the DAO itself, since intramuscular injection can cause pain and palpable nodules due to repetitive product compression.

In patients with milder wrinkles, only superficial filling in the subcutaneous plane may be needed. In this case, a filler with lower elasticity and a lower G prime is preferred. In others, deeper filling beneath the DAO may be desirable, using a filler with a higher G prime. Ultrasound can assist in selecting the optimal plane for each type of product. In patients with more pronounced wrinkles in this area, both the subcutaneous and submuscular planes may be treated, in what one of the authors, R. Vasconcelos-Berg, has termed the “sandwich technique”, which will be described as follows:

**Subcutaneous filler placement with a blunt cannula**

**Figure 9:**
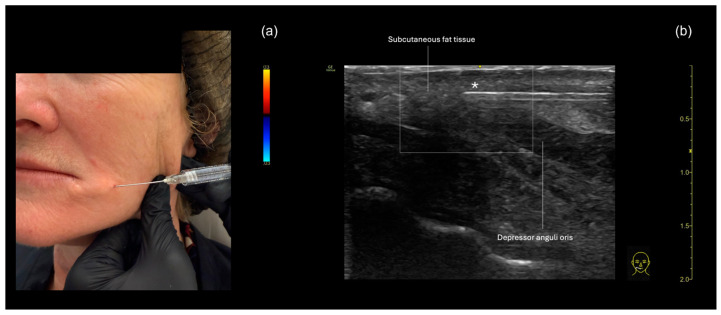
US-guided injection in the marionette line demonstrating subcutaneous injection with a cannula in the marionette region. (**a**) The 22 G cannula entry point is made laterally to the marionette line, and the triangular area is marked on the skin with a white pencil and filled. Due to the superficial nature of the filler, the contour of the cannula can be seen under the skin. The chosen filler should have a low G prime. (**b**) The ultrasound image shows the cannula in the subcutaneous plane, above the DAO muscle. The technique used is “scan while injecting”.

Since the inferior labial artery is often present in this region, we prefer to approach the marionette line area using a 22 G blunt cannula. The preferred entry point is lateral to the marionette line, positioning the cannula perpendicular to it (Figure 9a). However, an entry point in the pre-jowl region may also be used. For superficial filling, the cannula should be visualized below the skin and above the DAO (Figure 9b). The “scan while injecting” technique is employed in this approach.

**Submuscular filler placement with a blunt cannula**

**Figure 10:**
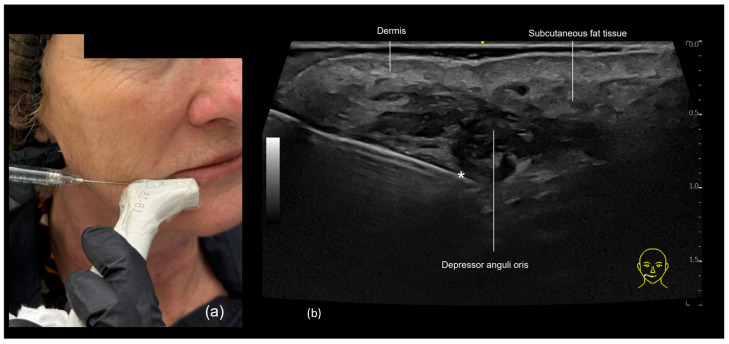
A US-guided injection in the marionette line demonstrating submuscular injection. (**a**) The 22 G cannula entry point is made laterally to the marionette line, and the triangular area is marked on the skin with a white pencil and filled. Since the filler will be injected at a deeper level, it is typically not possible to clearly visualize the contour of the cannula under the skin. (**b**) The cannula (*) is positioned in the submuscular plane, below the depressor anguli oris (DAO) muscle. The technique used is “scan while injecting”.

As with the subcutaneous technique, the submuscular technique is also performed using a cannula, utilizing the same entry point. However, it is essential to ensure that the cannula tip is positioned below the DAO and not in contact with the facial artery (Figure 10).

### 3.3. Lips

#### 3.3.1. Sonographic Anatomy

The lips are among the most commonly treated areas in esthetic procedures due to their central and prominent position on the face.

Externally, the lips consist of two primary sections: the cutaneous portion (or cutaneous lip) and the red portion (vermilion). In the upper lip, the cutaneous portion extends from the base of the nose to the vermilion border. The lower boundary of the cutaneous lip is defined by the mentolabial crease [14].

**Figure 11:**
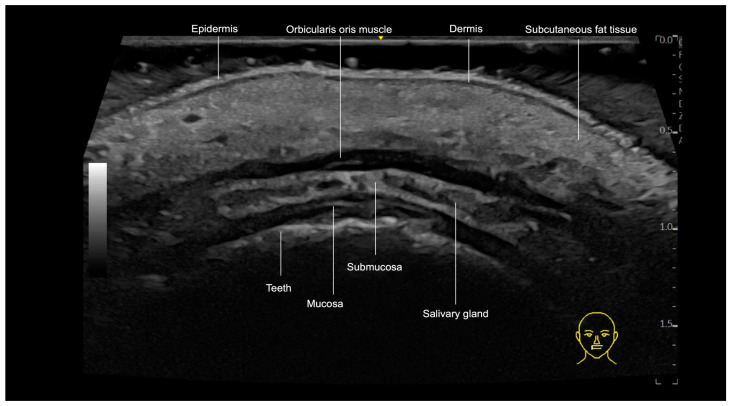
An ultrasound image of the cutaneous lip, transverse view, obtained with an 18 MHz probe. Note that in the cutaneous portion of the lip, the orbicularis oris muscle is present as a single layer, as it does not fold upon itself in this region, unlike in the vermilion of the lip.

**Figure 12:**
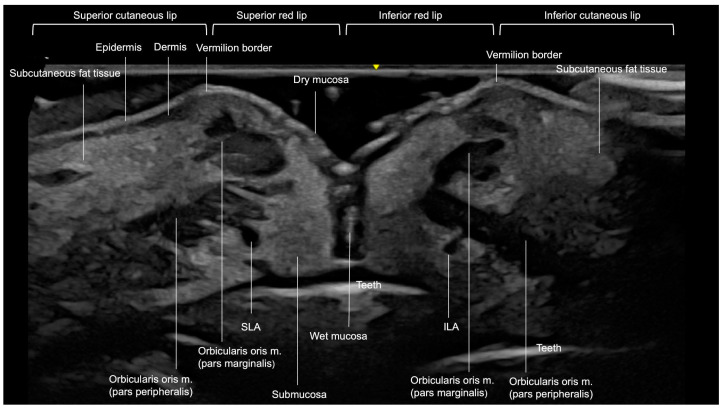
US of layers of lips, longitudinal view, 18 MHz probe. SLA: Superior labial artery, ILA: inferior labial artery.

Each anatomical layer of the lips exhibits distinct characteristics on ultrasound imaging (Figure 11 and Figure 12). The outermost layer, the epidermis, is visualized as a hyperechoic line. Directly beneath the epidermis, the dermis appears as a slightly hypoechoic layer, followed by the subcutaneous fat tissue. In the lips, this subcutaneous fat layer demonstrates increased echogenicity, attributed to its higher density of fibrous components. The next layer is the orbicularis oris muscle (OOM), which exhibits distinct ultrasound characteristics depending on its location. In the cutaneous lip (Figure 11), the OOM appears as a single hypoechoic band, while in the red lip (Figure 12), it presents as two hypoechoic bands due to its curved structure. On ultrasound, these two main parts are identifiable: the pars marginalis, which is the most superficial or external layer, and the pars peripheralis, situated deeper and closer to the submucosa.

Minor salivary glands are hypoechoic, rounded structures situated in the hyperechoic submucosa layer, deep to the pars peripheralis of the OOM (Figure 11).

The interface between the mucosa and the oral vestibule is visualized as a hyperechoic line on ultrasound. Beneath this line, the teeth are seen as prominent hyperechoic structures, representing the deepest visible layer on ultrasound.

The primary arterial supply to the lips originates from the superior and inferior labial arteries, which are branches of the facial artery arising from the lateral deep aspects of the lips. These arteries can be identified using ultrasound in various planes. Research by Cotofana et al. [14] indicated that the labial arteries most commonly reside in the submucosa (58.5%), followed by intramuscular (36.2%) and subcutaneous (5.3%) locations. More recent findings by Kim [15] show that the labial arteries course from a deeper plane (submucosal) laterally to a more superficial subcutaneous plane in the dry mucosa centrally.

**Figure 13:**
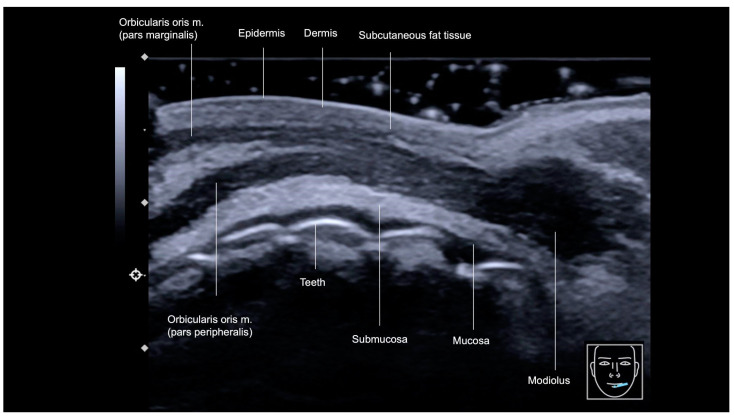
Ultrasound image of the transition between the corner of the mouth (modiolus region) and the lateral portion of the left lower lip. Transverse view, obtained with an 18 MHz probe. Note that in the vermilion region of the lip, both parts of the *orbicularis oris* muscle can be visualized.

The modiolus, a fibromuscular structure located lateral to the oral commissure, serves as a pivotal attachment point for several muscles, playing a crucial role in the complex movements of the mouth (Figure 13).

#### 3.3.2. Ultrasound-Guided Filling Techniques of the Lips

The choice of the location and depth of hyaluronic acid deposition is related to the treatment goal.

**Filling of the Cutaneous Lip**

In general, the filling of the cutaneous lip area aims to restore volume lost due to the aging process or to fill transverse wrinkles, contributing to the rejuvenation of this region [16].

**Figure 14:**
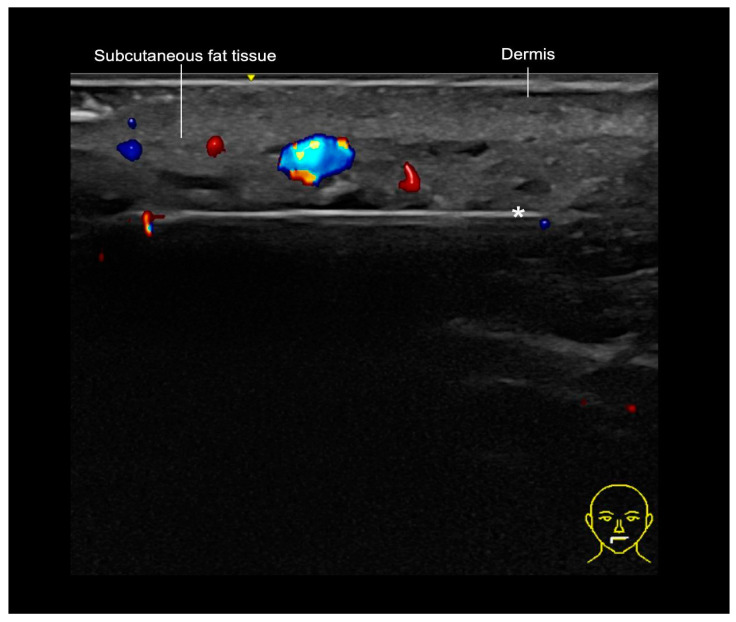
A Doppler US-guided injection of the cutaneous lip with a cannula (*) outside vessels using the “scan while injecting” technique.

Homogeneous subcutaneous or submuscular injections are used to restore volume lost with aging. A cannula is typically employed to evenly distribute a low G prime filler across areas of volume loss (Figure 14). In some cases, this procedure may result in mild lip eversion, reversing the inward rolling effect caused by age-related volume depletion.

This region can also be treated superficially, with injections placed perpendicularly to the vermilion border at multiple points beneath the so-called barcode lines. In this case, a low G prime filler is used to prevent excessive volumization, and a needle is typically employed in the subdermal layer [17].

**Filling the vermilion of the lip**

The red lip region can be treated for a variety of purposes, and numerous techniques have been described to modify lip shape, improve symmetry, enhance beauty, and rejuvenate the lips [18,19].

- In general, lip filler techniques can be categorized based on the following:
- Injection method: cannula or needle;
- Injection depth: superficial (below the dry mucosa) or deep (submuscular);

Surface anatomy considerations: targeting the lip contour, Cupid’s bow, lip tubercles, distal third of the lips, or oral commissures.

**Figure 15:**
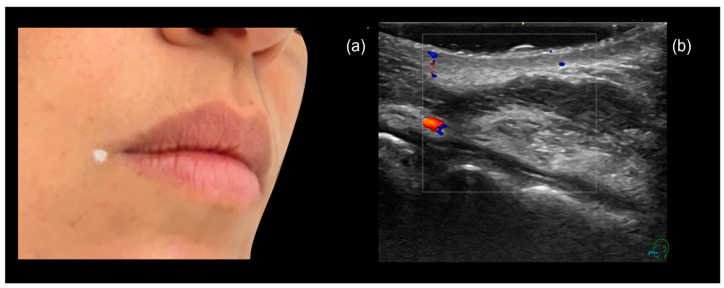
(**a**) Defining the cannula entry point prior to injection. (**b**) A sonographic image of the designated entry point, confirming the absence of relevant vascular structures.

When using a cannula, the entry point is typically placed laterally at the oral commissure. Occasionally, the injector may prefer to insert the cannula at the vermilion border, a few millimeters medially to the commissure. However, in the upper lip, this area is where the labial artery usually emerges from the facial artery and enters the lip [20]. To minimize the risk of intra-arterial injection, we recommend marking the puncture site with a pencil, and performing pre-procedural ultrasound scanning (‘scan before injecting’) can help ensure a safe entry point (Figure 15a).

**Figure 16:**
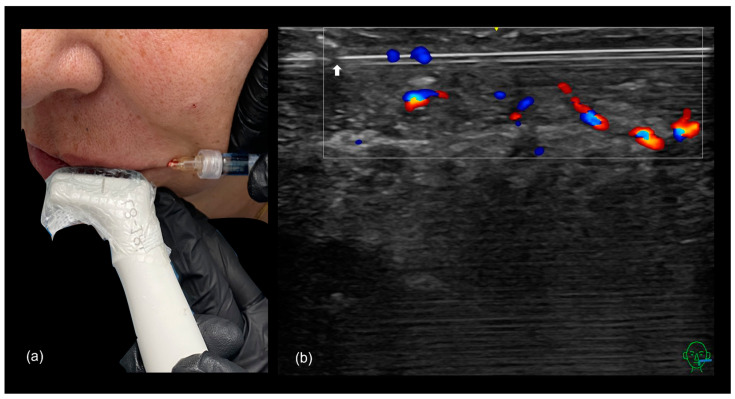
(**a**) Lip augmentation using a cannula with the “scan while injecting” technique. A 25 G cannula is positioned in-plane with the transducer. (**b**) Doppler ultrasound shows the cannula (the white arrow pointing to the cannula tip) positioned superficially and parallel to the superior labial artery (depicted in red and blue).

The “scan while injecting” technique (Figure 16) can be used during cannula-based treatments to avoid labial artery injury and to precisely define the injection plane, ensuring placement either above, below, or focusing on the intermediate region between the two parts of the orbicularis oris muscle.

Some authors [21] advocate for intramuscular filler injections to increase the volume of the region. However, these publications are made without the aid of imaging studies to confirm the exact location of the filler, making it impossible to determine whether the volumizing effect truly resulted from an intramuscular injection.

**Figure 17:**
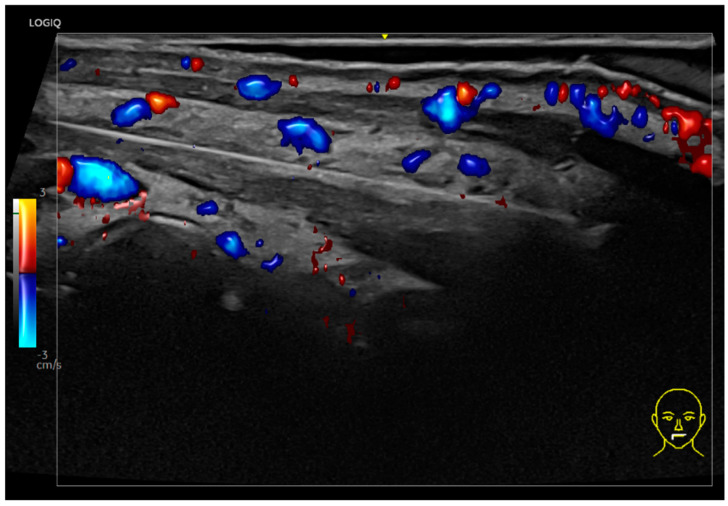
Volumization of the lip vermilion using a 25 G cannula in the submuscular plane. The “scan while injecting” technique is employed, with the cannula positioned in-plane.

Deep filler injections are primarily intended to restore volume in the treated area. Since the labial artery is most commonly located in the submuscular layer, this approach carries a higher risk of vascular occlusion. For this reason, cannula injections are often preferred in these cases, particularly when combined with ultrasound guidance (“scan while injecting”) to enhance safety (Figure 17).

If needle injections are required for deep injections, a possible approach is to measure the depth of the labial artery in the treatment area before injecting, avoiding needle use at the same depth. It is important to note that without real-time ultrasound guidance, it is impossible to determine the precise needle depth during the procedure.

**Figure 18:**
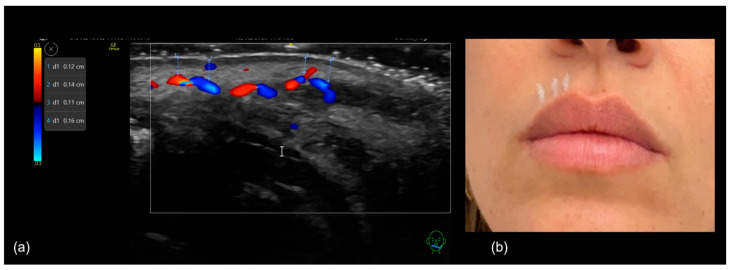
Pre-injection scanning of the labial artery for superficial needle injection. Based on the Doppler ultrasound image (**a**), the skin is marked at points where the labial artery is most superficial (**b**). In the case of the patient above, the shortest distance between the skin and the labial artery was 0.11 cm (**a**). Therefore, any superficial injection using a needle should not exceed this depth, especially at the marked points on the skin. To ensure accurate measurement of skin structures without distortion caused by pressure, it is crucial to maintain a sufficient amount of gel between the transducer and the skin while applying minimal pressure during the ultrasound examination.

Measuring the arterial depth prior to injection can also be beneficial in superficial filler applications, as it allows practitioners to determine the maximum safe depth for needle insertion (Figure 18).

It is well known that the depth of the labial artery is not uniform along the length of the lips. Therefore, it is essential to measure the arterial depth at each specific injection site to enhance safety and procedural accuracy.

### 3.4. Chin

#### 3.4.1. Sonographic Anatomy of the Chin

The chin, or mentum, is a complex anatomical region composed of skin, a fibrofatty layer, muscles, and multiple neurovascular structures and networks. It is bordered by the lower lip, the pre-jowl sulci bilaterally, and the inferior border of the mandible.

The chin is crucial for facial balance, supporting the lower third of the face, contributing to the profile, and guiding perceptions of youth and attractiveness. Fillers are often used to enhance the chin, and are injected near the pogonion and along the menton, to achieve better facial harmony and proportion.

On ultrasound, dense subcutaneous tissue and a complex convex shape of the chin can present difficulties in scanning and require attention to the proper position of the probe and plenty of ultrasound gel.

**Figure 19:**
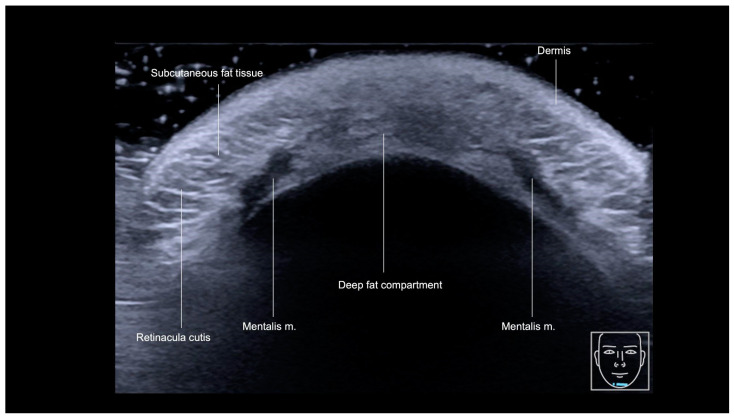
Gray-scale US of chin, transverse view, 18 MHz probe.

Starting superficially, layers show as the hyperechoic epidermis, hypoechoic upper dermis, and hyperechoic lower dermis, extending deeper as the hyperechoic, poorly defined, fibrofatty layer, down to two symmetric hypoechoic bellies of the mentalis muscle. In between these bellies there is a hyperechoic deep chin fat pad (Figure 19). We may also see medial extensions of the DAO and depressor labii inferioris muscles [22,23].

**Figure 20:**
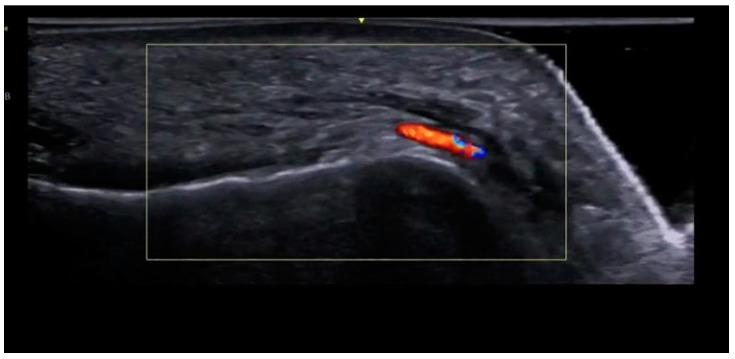
A paramedian sagittal view of the chin, with a color Doppler view of the submental artery under the mentalis muscle (GE Healthcare, Waukesha, WI, USA Venue Fit, L4-20t-RS probe).

The vascular anatomy of the chin is notable for its variability [23]. Color Doppler mapping reveals submental arteries—usually just to the side of the midline, but occasionally crossing midline—mental arteries, and inferior labial or labiomental arteries (Figure 20), with multiple anastomoses in between these vessels.

#### 3.4.2. Ultrasound-Guided Filling Techniques of the Chin

The chin is crucial for facial balance—it supports the lower third of the face, shapes the profile, and influences perceptions of youth and attractiveness. Fillers are often used to enhance the chin by injecting near the pogonion and along the menton, thereby improving overall facial harmony and proportions [5].

When volumizing the chin area, it is important to consider the local vascular structures. Due to the presence of multiple arterial structures in this relatively small area [22], vascular occlusion complications can occur.

**Figure 21:**
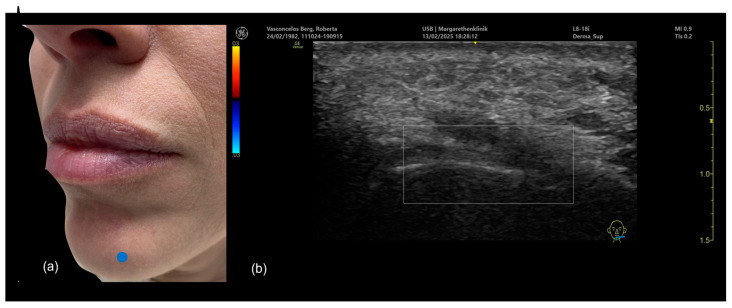
(**a**) Marking the skin and scanning the points to be injected with a needle for chin projection. The goal at these points is to fill the supraperiosteal plane with a high G prime filler. Therefore, it is important to evaluate this plane using Doppler color imaging (**b**).

Vascular mapping before injection (“scan before injecting”) is routinely used when performing needle injections on the bone along the menton and pogonion (Figure 21). It is essential to identify and avoid the submental arteries that can be located in the midline, and to scan for the midline mandibular/inferior alveolar canal with a perforating median artery, which may communicate with the lingual circulation. Avoiding these arteries is crucial to prevent skin and tongue necrosis.

For the central deep compartment of the chin, a deep injection using a 25 G or 22 G cannula under real-time ultrasound guidance (“scan while injecting”) is indicated if a midline perforating artery is detected during pre-scanning, and may be necessary to avoid the mental foramina and mental arteries.

**Figure 22:**
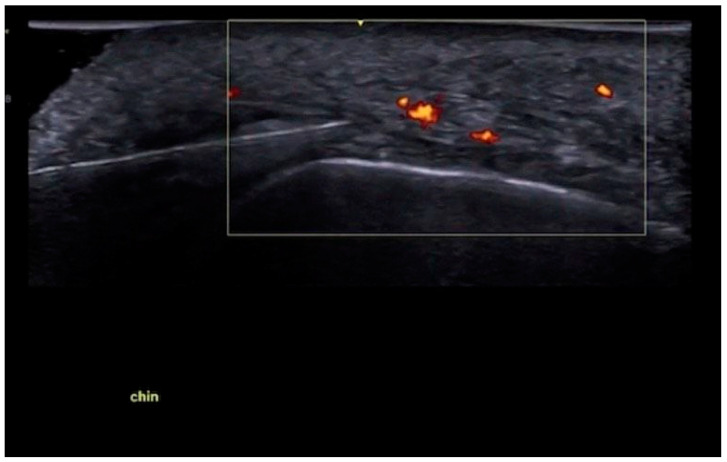
Chin filler injection using a 25 G cannula. The US-Doppler image shows the cannula in the deep fat plane.

Some injectors may prefer to project the submuscular plane below the mentalis muscle using a cannula. It can also be used to fill the more superficial subcutaneous areas, such as the labiomental sulcus or the pre-jowl area. In all these cases, the ‘scan while injecting’ technique can be applied (Figure 22).

## 4. Discussion

This article represents the final installment in a three-part series [8,9] focused on the lower third of the face. It outlines clear, reproducible techniques for performing ultrasound-guided filler injections in this region.

The lower face is a common target for esthetic treatments, particularly for repositioning anatomical structures affected by age-related volume loss, such as the pre-jowl area, labiomental folds, and lips. The anatomy of this region has been extensively documented in the literature [5,21,22], though it can exhibit significant variability. The use of high-resolution ultrasound has become increasingly prevalent in filler procedures, offering enhanced accuracy for identifying anatomical variations, avoiding critical structures, and minimizing potential complications.

The mandibular region, with its proximity to key anatomical structures such as the facial artery, parotid gland, and masseter muscle, presents particular challenges in esthetic procedures. The parotid gland, located just beneath the skin in the preauricular area, increases the risk of complications. In this context, ultrasound guidance plays a pivotal role in preventing unnecessary injections into this gland, ensuring a safer treatment approach. Additionally, inadvertent injections into the masseter muscle can lead to pain and restricted jaw movement. By using ultrasound to assess muscle depth at the injection site, injectors can select the appropriate needle length and reduce the risk of injecting into the muscle. Continuous monitoring of the injection site via ultrasound ensures accurate filler placement, minimizes complications, and improves esthetic outcomes, especially in areas like the jawline and pre-jowl region, which are prone to age-related volume loss and changes.

For marionette lines, ultrasound guidance further enhances the precision and safety of filler injections in this anatomically complex area. With aging, these folds become more pronounced, and ultrasound enables accurate identification of key structures such as the depressor anguli oris muscle and the inferior labial artery, helping to avoid complications such as intramuscular injections and vascular injury. The “sandwich technique,” which targets both the subcutaneous and submuscular planes, provides a comprehensive approach for treating deeper wrinkles. Combining ultrasound with careful technique is essential for achieving optimal results while minimizing risks in esthetic treatments for the lower face.

When addressing the lips, practitioners must be mindful of the significant anatomical variability of the superior and inferior labial arteries. We strongly recommend using ultrasound guidance to verify the precise injection plane, particularly when using sharp needles in this sensitive area.

The chin plays a critical role in facial harmony, and fillers are frequently used to enhance its profile. Ultrasound guidance is essential to avoid vascular complications, such as occlusion of the submental and mental arteries. The “scan before injecting” and “scan while injecting” techniques ensure precise and safe injections, leading to optimal results.

Inadvertent intra-arterial injections of hyaluronic acid can cause severe complications, including vascular occlusion, necrosis, and even blindness [24,25]. This article emphasizes the importance of accurately determining the artery’s location before or during the procedure to avoid unintentional injection into the vessel.

The main limitation of this article is that it reflects the experience of a single group of experienced injectors. However, readers can adapt the use of ultrasound to other filler techniques as needed, based on individual treatment goals. In the future, more widespread use of ultrasound among injectors is expected to foster the development of new application strategies, making the method even more reproducible and broadly applicable.

The use of ultrasound to guide injectable procedures is still significantly limited by the need for additional training among injectors and the high cost of equipment. Looking ahead, we anticipate that ultrasound-guided injection techniques will continue to evolve, becoming increasingly refined and precise. Ultrasound devices are also expected to become more portable, lightweight, and affordable, which will further support their integration into everyday clinical practice. These advancements will enable more targeted treatment of specific anatomical regions, optimizing esthetic outcomes while minimizing the risk of complications. As a result, the use of ultrasound in esthetic procedures is likely to become more widespread and standardized.

## 5. Conclusions

Ultrasound-guided techniques for lower face filler injections are an effective tool for reducing complications and improving esthetic outcomes. These guidelines provide key concepts for injectors seeking to integrate this technology into their practice. By adopting these approaches, practitioners can achieve safer and more precise results, ultimately enhancing patient satisfaction and treatment success.

## Data Availability

Data sharing is not applicable to this article.

## References

[1] Salinas C.A., Liu A., Sharaf B.A. (2024). Analysis of Sexual Dimorphic Features of the Jawline and Chin in White Celebrity Faces. J. Craniofac. Surg..

[2] de Maio M. (2015). Ethnic and Gender Considerations in the Use of Facial Injectables: Male Patients. Plast. Reconstr. Surg..

[3] Toledo Avelar L.E., Cardoso M.A., Santos Bordoni L., de Miranda Avelar L., de Miranda Avelar J.V. (2017). Aging and Sexual Differences of the Human Skull. Plast. Reconstr. Surg.—Glob. Open.

[4] Keaney T.C., Anolik R., Braz A., Eidelman M., Eviatar J.A., Green J.B., Jones D.H., Narurkar V.A., Rossi A.M., Gallagher C.J. (2018). The Male Aesthetic Patient: Facial Anatomy, Concepts of Attractiveness, and Treatment Patterns. J. Drugs Dermatol..

[5] Braz A., Eduardo C.C.P. (2020). Reshaping the Lower Face Using Injectable Fillers. Indian J. Plast. Surg..

[6] de Maio M. (2018). Myomodulation with Injectable Fillers: An Innovative Approach to Addressing Facial Muscle Movement. Aesthet. Plast. Surg..

[7] Coimbra D.D., Stefanello B. (2023). Myomodulation with Facial Fillers: A Comprehensive Technical Guide and Retrospective Case Series. Aesthet. Plast. Surg..

[8] Sigrist R., Desyatnikova S., Chammas M.C., Vasconcelos-Berg R. (2024). Best Practices for the Use of High-Frequency Ultrasound to Guide Aesthetic Filler Injections—Part 1: Upper Third of the Face. Diagnostics.

[9] Vasconcelos-Berg R., Desyatnikova S., Bonavia P., Chammas M.C., Navarini A., Sigrist R. (2024). Best Practices for the Use of High-Frequency Ultrasound to Guide Aesthetic Filler Injections—Part 2: Middle Third of the Face, Nose, and Tear Troughs. Diagnostics.

[10] Vasconcelos-Berg R., Izidoro J.F., Wenz F., Müller A., Navarini A.A., Sigrist R.M.S. (2023). Doppler Ultrasound-Guided Filler Injections: Useful Tips to Integrate Ultrasound in Daily Practice. Aesthet. Surg. J..

[11] Kochhar A., Larian B., Azizzadeh B. (2016). Facial Nerve and Parotid Gland Anatomy. Otolaryngol. Clin. N. Am..

[12] Schelke L., Schoonen T., Velthuis P.J. (2023). Filler Injections in the Pre-Auricular Space: Be Aware of the Parotid Gland. J. Cosmet. Dermatol..

[13] Hong G.W., Kim S.B., Park S.Y., Wan J., Yi K.H. (2024). Why Do Marionette Lines Appear? Exploring the Anatomical Perspectives and Role of Thread-Based Interventions. Ski. Res. Technol..

[14] Cotofana S., Alfertshofer M., Schenck T.L., Bertucci V., Beleznay K., Ascher B., Lachmann N., Green J.B., Swift A., Frank K. (2020). Anatomy of the Superior and Inferior Labial Arteries Revised: An Ultrasound Investigation and Implication for Lip Volumization. Aesthet. Surg. J..

[15] Kim J.S. (2024). 9-Point Injection Technique for Lip Augmentation and Lip Corner Lifting Using Sonographic Imaging of the Labial Artery Pathway. Aesthet. Surg. J..

[16] Rohrich R.J., Pessa J.E. (2009). The Anatomy and Clinical Implications of Perioral Submuscular Fat. Plast. Reconstr. Surg..

[17] Baudoin J., Meuli J.N., di Summa P.G., Watfa W., Raffoul W. (2019). A Comprehensive Guide to Upper Lip Aesthetic Rejuvenation. J. Cosmet. Dermatol..

[18] Cooper H., Gray T., Fronek L., Witfill K. (2023). Lip Augmentation with Hyaluronic Acid Fillers: A Review of Considerations and Techniques. J. Drugs Dermatol..

[19] Stojanovič L., Majdič N. (2019). Effectiveness and Safety of Hyaluronic Acid Fillers Used to Enhance Overall Lip Fullness: A Systematic Review of Clinical Studies. J. Cosmet. Dermatol..

[20] Tansatit T., Apinuntrum P., Phetudom T. (2014). A Typical Pattern of the Labial Arteries with Implication for Lip Augmentation with Injectable Fillers. Aesthet. Plast. Surg..

[21] Trévidic P., Criollo-Lamilla G. (2020). French Kiss Technique: An Anatomical Study and Description of a New Method for Safe Lip Eversion. Dermatol. Surg..

[22] Lee H.J., Won S.Y., O J., Hu K.S., Mun S.Y., Yang H.M., Kim H.J. (2018). The Facial Artery: A Comprehensive Anatomical Review. Clin. Anat..

[23] Iwanaga J., Matsushita Y., Decater T., Ibaragi S., Tubbs R.S. (2021). Mandibular Canal vs. Inferior Alveolar Canal: Evidence-Based Terminology Analysis. Clin. Anat..

[24] Quach B., Clevens R.A. (2024). Complications of Injectables. Atlas Oral Maxillofac. Surg. Clin. N. Am..

[25] Kyriazidis I., Spyropoulou G.-A., Zambacos G., Tagka A., Rakhorst H.A., Gasteratos K., Berner J.E., Mandrekas A. (2024). Adverse Events Associated with Hyaluronic Acid Filler Injection for Non-Surgical Facial Aesthetics: A Systematic Review of High Level of Evidence Studies. Aesthet. Plast. Surg..

